# Mitophagy in hypertension-mediated organ damage

**DOI:** 10.3389/fcvm.2023.1309863

**Published:** 2024-01-04

**Authors:** Yulong Ma, Xunjie Zhou, Mingtai Gui, Lei Yao, Jianhua Li, Xiaozhe Chen, Mingzhu Wang, Bo Lu, Deyu Fu

**Affiliations:** Department of Cardiology, Yueyang Hospital of Integrated Traditional Chinese and Western Medicine, Shanghai University of Traditional Chinese Medicine, Shanghai, China

**Keywords:** hypertension, organ damage, mitochondria, mitochondrial quality control, mitophagy

## Abstract

Hypertension constitutes a pervasive chronic ailment on a global scale, frequently inflicting damage upon vital organs, such as the heart, blood vessels, kidneys, brain, and others. And this is a complex clinical dilemma that requires immediate attention. The mitochondria assume a crucial function in the generation of energy, and it is of utmost importance to eliminate any malfunctioning or surplus mitochondria to uphold intracellular homeostasis. Mitophagy is considered a classic example of selective autophagy, an important component of mitochondrial quality control, and is closely associated with many physiological and pathological processes. The ubiquitin-dependent pathway, facilitated by PINK1/Parkin, along with the ubiquitin-independent pathway, orchestrated by receptor proteins such as BNIP3, NIX, and FUNDC1, represent the extensively investigated mechanisms underlying mitophagy. In recent years, research has increasingly shown that mitophagy plays an important role in organ damage associated with hypertension. Exploring the molecular mechanisms of mitophagy in hypertension-mediated organ damage could represent a critical avenue for future research in the development of innovative therapeutic modalities. Therefore, this article provides a comprehensive review of the impact of mitophagy on organ damage due to hypertension.

## 1 Introduction

Hypertension has emerged as the predominant risk factor for mortality in the world (1). Epidemiological surveys indicate that the worldwide incidence of hypertension has surged to encompass 1.3 billion individuals (2). In 2019, hypertension affected 32% of men and 34% of women within the cohort aged 30 to 79 globally (3). Between 2017 and 2020, the National Health and Nutrition Examination Survey revealed a high hypertension prevalence of about 46.7% among adults aged 20 years and older. The rates of hypertension awareness, treatment, and control were 62%, 52.6%, and 25.7% (4, 5). It is estimated that annually, elevated blood pressure results in the deaths of between 7.7 and 10.4 million individuals (6). A prospective study revealed that for each increase of 20 mmHg in systolic blood pressure and 10 mmHg in diastolic blood pressure, the risk of developing ischemic heart disease and stroke doubled. The findings indicate a strong correlation between blood pressure levels and subsequent cardiovascular events (7). Hypertension-mediated organ damage (HMOD) including stroke, ischemic heart disease, kidney disease, and other vascular conditions, is responsible for causing 85 million deaths globally (8, 9). The treatment and management of hypertension pose a significant burden on the healthcare system worldwide. Despite the extensive use of efficient antihypertensive medications, combatting HMOD remains an urgent medical challenge.

Mitochondria are specialized double-membrane organelles that originated from the engulfment of *α*-amoebae by eukaryotic progenitor cells (10). Mitochondria function as metabolic signaling centers and energy production sites, supplying the necessary biochemical reactions for cellular activity (11). Their role in maintaining cell survival, death, and metabolic homeostasis is paramount (12). Maintaining intracellular mitochondrial homeostasis necessitates a quality control system comprising mitochondrial biogenesis, mitochondrial dynamics, mitophagy, and mitolysosome exocytosis (13, 14). Mitochondria are integral organelles in cardiomyocytes that perform vital functions necessary for maintaining appropriate myocardial activity (15). Mitophagy contributes to intracellular homeostasis by eliminating damaged and excess mitochondria (16). Under typical circumstances, mitophagy supports the preservation of cardiomyocyte energy metabolism homeostasis by eliminating unhealthy mitochondria (17). When mitophagy is either insufficient or excessive, it causes imbalanced mitochondrial homeostasis, which subsequently initiates a range of diseases. Mitophagy can have a positive or negative impact on cardiovascular disease, contingent on the preservation of mitochondrial homeostasis (18). Therefore, this review focus on the correlation between faulty mitophagy, which encompasses inadequate and excessive mitophagy, and multiple HMOD.

## 2 Overview of mitophagy

**Figure 1:**
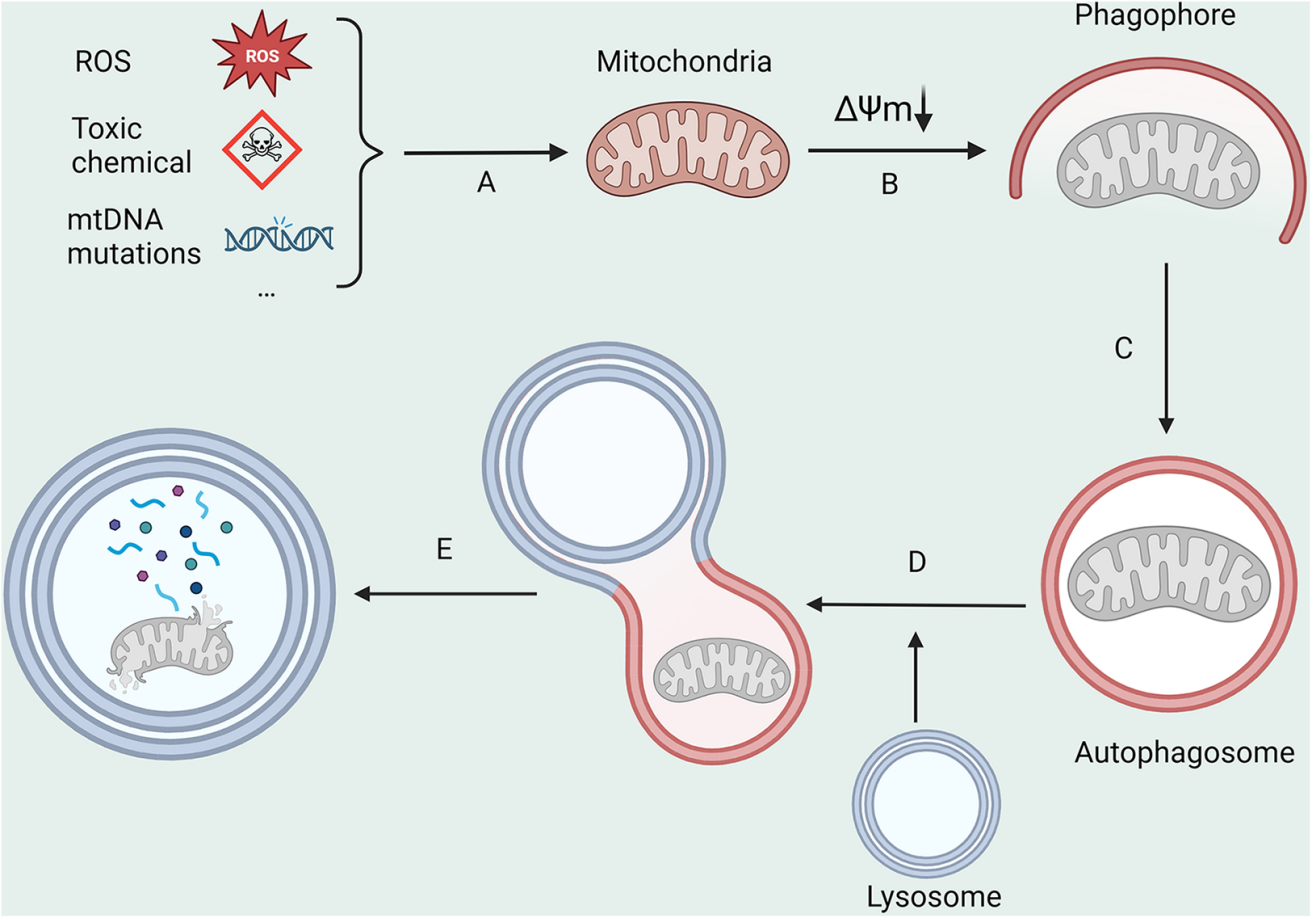
The main processes in mitophagy. (**A**) ROS, toxic chemicals, and mitochondrial DNA mutations lead to a decrease in mitochondrial membrane potential; (**B,C**) Damaged mitochondria are encapsulated by autophagosomes; (**D**) Autophagosomes fuse with lysosomes; (**E**) Mitochondrial is degraded by lysosomes. All figures were created with BioRender.com and are licensed for use.

Mitochondria, often referred to as the body's “energy production center”, are susceptible to substantial levels of exposure to reactive oxygen species (ROS) due to their unique functions (19). This can easily cause mutations in mitochondrial DNA (mtDNA) and result in structural and functional changes in proteins, leading to mitochondrial harm. Mitophagy (Figure 1), the process responsible for the degradation of dysfunctional mitochondria, is crucial in maintaining intracellular homeostasis (20). The primary modalities that govern mitophagy encompass ubiquitin-dependent, receptor-dependent, and other pathways (21).

### 2.1 Ubiquitin-mediated pathway

In the realm of ubiquitin-dependent mitophagy, the PTEN-induced putative kinase protein 1 (PINK1)-Parkin pathway stands as an exemplar (20). PINK1 is a serine protease located upstream of Parkin. Normally, PINK1 is transported inside the mitochondria and eventually degraded by the proteasome (22–24). The amount of Parkin in the cytoplasm is minimal. PINK1 stabilizes on the out mitochondrial membrane (OMM) when there is a loss of mitochondrial membrane potential or accumulation of misfolded proteins (25, 26). PINK1 phosphorylates ubiquitin at Ser65, which is a critical step in activating Parkin. This phosphorylation is essential for Parkin to function properly (27–29). However, the phosphorylation of Parkin alone is inadequate to bestow activity. Activated Parkin subsequently forms ubiquitin chains on several proteins in the OMM, ultimately attracting autophagy receptors that bind to ubiquitin (22). A set of receptor proteins, including Sequestosome-1 (SQSTM1 or p62), Optineurin (OPTN), Next to BRCA1 gene 1 protein (NBR1), Calcium-binding and coiled-coil domain-containing protein 2 (NDP52 or CALCOCO2), and Tax1-binding protein 1 (TAX1BP1), interact with microtubule-associated protein 1A/1B-light chain 3 (LC3), thereby initiating the mitophagy process (30, 31). Notably, phosphorylation of ubiquitin induced by PINK1 serves as a signal for mitophagy, which is subsequently amplified by Parkin (26, 32). The deubiquitinase USP30 has demonstrated its ability to remove ubiquitin from depolarized mitochondria and hinder mitophagy (33).

Moreover, multiple signaling pathways influence mitophagy by modulating the expression of PINK1/Parkin. Autophagy and beclin 1 regulator 1 (AMBRA1) plays a important role in mitophagy, regulating this process through its interaction with ATPase family AAA domain containing 3A (ATAD3A) and by strengthening the stability of PINK1 (34). Targeting ATAD3A also overcomes resistance to chemoimmunotherapy by redirecting PD-L1 to mitochondria (35). LncRNA H19 downregulates mitophagy by limiting the translation of Pink1, thereby attenuating cardiac injury induced by obesity (36). Spliced X-box binding protein 1 (XBP1s) regulates mitophagy by interacting with PINK1, an interaction which is transcription- and phosphorylation-dependent (37). In addition to regulating mitophagy, PINK1 plays other roles, including inhibiting tumor growth through and reducing production of acetyl coenzyme A (38).

### 2.2 Ubiquitin-independent pathway

Mitophagy via the ubiquitin-independent pathway is also commonly referred to as receptor-dependent mitophagy. Unlike mitophagy facilitated by the PINK1/Parkin pathway, the receptor-mediated pathway does not necessitate ubiquitin participation. The OMM contains a range of receptor proteins which have the ability to bind to LC3. For example, BCL2/adenovirus E1B 19 kDa protein-interacting protein 3 (BNIP3), BNIP3-Like (BNIP3l or NIX), and FUN14 domain-containing protein 1 (FUNDC1) can interact directly with LC3, resulting in recognition by the autophagosome. This process is the driving mechanism behind the direction of mitophagy.

#### 2.2.1 BNIP3, NIX

BCL2 family members are categorized based on the presence of one or more BCL2 homologous structural domains (39). BNIP3 and BNIP3l, which have structural domain homology to BCL2 in BH3, are proteins produced by the BNIP3 gene located on chromosome 10q26.3. They have a molecular weight of 21.5 kDa and a peptide chain comprising 194 amino acids. These proteins play significant roles in the processes of autophagy (40). BNIP3 protein is expressed at low levels in cells and tissues under normal circumstances, including skeletal muscle, brain, and heart (40, 41). Under hypoxic conditions, the BNIP3 protein undergoes upregulation and becomes anchored to the OMM through its C-terminal transmembrane structural domain. This exposes the N-terminal structural domain to the cytoplasm (42). Like other mitophagy receptor proteins, BNIP3 contains an LC3-interacting region (LIR) motif at its N-terminus. The phosphorylation of the two tandem serine residues, Ser 34 and Ser 35, that are near the LIR motif, stabilizes the NIX-LC3 interaction, leading to the promotion of mitophagy (43). Furthermore, BNIP3 plays a crucial role in regulating ROS production during mitophagy. It has been reported that during hypoxia, hypoxia-inducible factor-1α (HIF1α) promotes BNIP3 expression, which limits ROS production through mitophagy (44, 45). Increased HIF1α expression, in contrast, led to an upregulation of mitochondrial ROS levels in BNIP3-KO tumor cells (46). Furthermore, mitophagy mediated by HIF1α/BNIP3 in renal tubular cells protects against acute kidney injury induced by reperfusion after ischemia by inhibiting apoptosis and ROS generation (47). Similar to BNIP3, the dimerization of NIX, specifically the phosphorylation of its C-terminal region, is crucial for the targeted autophagy in the mitochondria (48). NIX participates in the clearance of mitochondria in the course of reticulocyte maturation (49, 50). The SCF-FBXL4 complex, operating as a ubiquitin E3 ligase in the mitochondria, promotes the degradation of BNIP3 and NIX, resulting in the inhibition of mitophagy (51). However, BNIP3/NIX is not entirely independent of mitophagy mediated by PINK1/Parkin. BNIP3 interacts with PINK1, leading to an accumulation of PINK1 on the OMM. This, in turn, promotes the recruitment of Parkin to mitochondria (52). Inhibition of cyclin dependent kinase 9 (CDK9) inhibits PINK1/Parkin-mediated initiation of mitophagy through regulation of the SIRT1/FOXO3/BNIP3 axis (53). The ubiquitination of NIX by Parkin facilitates the binding of ubiquitin and LC3 through the selective autophagy junction protein NBR1. As a result, autophagosome wrapping around mitochondria is promoted (54).

#### 2.2.2 FUNDC1

FUNDC1 is situated within the OMM and is characterized by a canonical LIR motif, in addition to three transmembrane structural domains near its N-terminus (55). This protein serves as a receptor for mitophagy induced by hypoxia. Serine 13 and Tyrosine 18 residues of FUNDC1 can be dephosphorylated under hypoxic stress and in abnormal mitochondrial membrane potential situations. The LIR motif of FUNDC1 interacts hydrophobically with the Y and L pockets of the LC3 protein thereby inducing mitophagy (56, 57). FUNDC1-mediated mitophagy is tightly dependent on the interaction between typical LIR motifs and LC3 (58). In hypoxic conditions, the expression of FUNDC1 was decreased (55). In addition, uULK1 has the ability to phosphorylate FUNDC1, which activates mitophagy. During hypoxia or mitochondrial depolarization, ULK1 expression is induced, and it targets mitochondria to phosphorylate FUNDC1 at Ser17 (near the LIR motif). This leads to an enhanced interaction of FUNDC1 with LC3 (59).

#### 2.2.3 Other pathways

In addition to the mitophagy-mediated mechanisms mentioned earlier, alternative pathways exist for the elimination of dysfunctional mitochondria. Bcl-2-like protein 13 (Bcl2-L-13) is a single-channel membrane protein anchored to the OMM. It binds to LC3 through the LIR motif, recruiting the ULK1 complex to trigger mitophagy in Parkin-deficient cells (60). FK506-binding protein 8 (FKBP8) can induce mitophagy by interacting with LC3A through its LIR motifs (61). minichromosome maintenance complex component 8 (MCM8) initiates mitophagy by binding to LC3 via the LIR motif. Additionally, this type of mitophagy mediated by MCM8 operates independently of previously identified mechanisms (62). While in cells where Bcl2-L-13 is knocked down, there is a reduction in fission and mitophagy due to mitochondrial damage (63). Recent studies have shown that damaged mitochondria are eliminated through extracellular vesicles when lysosomal degradation is inhibited (64). Flunarizine induces the fusion of mitochondria and lysosomes resulting in the generation of a unique structure known as the mitolysosome. This phenomenon occurs through a direct mechanism (65). The mitolysosome is expelled to the extracellular compartment through cytokinesis dependent on VAMP2/ STX4 as vesicles, leading to a reduction in the total number of mitochondria within the cell. This approach works independently of autophagy protein 5 (ATG5) or ras-related protein 9 (RAB9)-mediated mitophagy (65). Migratory cells selectively eliminate impaired mitochondria using migratory bodies to maintain organismal balance, seamlessly linking mitochondrial balance to cell migration (66).

## 3 Mitophagy in hypertension-mediated organ damage

**Figure 2:**
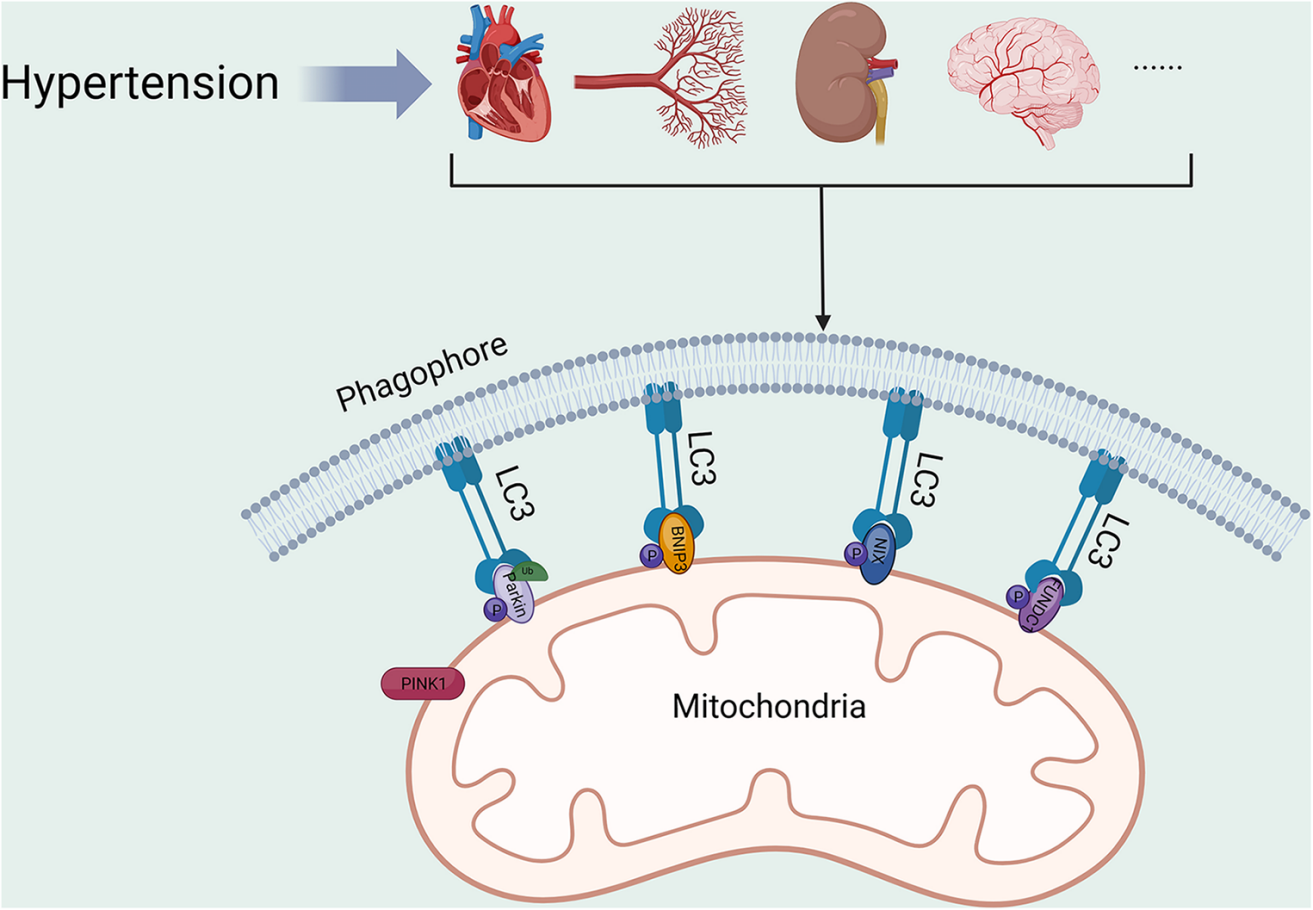
Mitophagy in HMOD. PINK1, PTEN-induced putative kinase protein 1; BNIP3, BCL2/adenovirus E1B 19 kDa protein-interacting protein 3; NIX, BCL2/adenovirus E1B 19 kDa protein-interacting protein 3-like; FUNDC1, FUN14 domain-containing protein 1; LC3, microtubule-associated protein 1A/1B-light chain 3. All figures were created with BioRender.com and are licensed for use.

Hypertension constitutes a substantial risk factor for various life-threatening diseases (67–69). Consequently, proper mitochondrial function is crucial to cardiovascular health. Alterations in mitochondrial function, structure and homeostasis can be commonly seen in organ tissues of patients with hypertension. Mitochondrial hyperacetylation leads to a cyclic relationship between metabolic disturbances and mitochondrial oxidative stress, which contributes to vascular dysfunction and hypertension (70). A systematic investigation involving 264 hypertensive patients discovered a connection between mitochondrial dysfunction and hypertension. Nevertheless, additional research is necessary to clarify the relationship between mt-DNA mutations and the development of hypertension (71). Abnormalities in mitochondrial function or structure have been discovered in numerous experimental hypertension models (72–74). Overexpression of DNA-damage regulated autophagy modulator 1 (DRAM1) was found to reduce oxidative stress, improve mitochondrial fusion and fission, and increase mitophagy in the placenta of pre-eclamptic mice. These improvements ultimately lead to a reduction in blood pressure (75). Mitophagy serves as a crucial element in HMOD (Figure 2, Table 1), being a component of the mitochondrial quality control system. Studies indicate that there is a correlation between mitophagy activity and hypertension, as genetic variants in the PARK2 gene, which encodes Parkin, have been found to elevate blood pressure level (88).

**Table 1:** The role of mitophagy in HMOD.

Damaged organ	Mechanisms	Effects on mitophagy	References
Cardiac	FoxP3 inhibits Parkin expression and downregulates excessive mitophagy by binding to ATF4 downstream sites or sequestering ATF4 in the nucleus, thereby ameliorating myocardial remodeling.	Inhibit	(76)
Cardiac	GSNOR promotes mitophagy through anti-denitrification mechanisms and inhibits cardiac hypertrophy.	Promote	(77)
Cardiac	SIRT3 promotes mitophagy and mitigates cardiac injury by upregulating PINK1/Parkin.	Promote	(78)
Cardiac	Valsartan inhibits excessive mitophagy and alleviates ventricular hypertrophy by modulating autophagy-related genes, such as Atg5.	Inhibit	(79)
Cardiac	Spermine reduces blood pressure and ameliorates cardiac hypertrophy in salt-sensitive hypertensive rats by enhancing mitophagy.	Promote	(80)
Vascular	Astaxanthin enhances mitophagy and enhances vascular remodeling by increasing PINK1/Parkin.	Promote	(81)
Vascular	The overexpression of SIRT3 enhances mitophagy to stimulate angiogenesis.	Promote	(78)
Kidney	Adiporon mitigates renal fibrosis in salt-sensitive hypertensive mice by enhancing autophagy.	Promote	(82)
Cerebrum	The activation of mitophagy reduces the risk of stroke in spontaneously hypertensive rats on a high-salt diet.	Promote	(83)
Cerebrum	Defective mitophagy leads to neuronal cell injury during chronic hypoxia.	Promote	(84)
Cerebrum	The activation of mitophagy eliminates damaged mitochondria from neurons and safeguards their integrity.	Promote	(85)
Cerebrum	SYNJ2BP and SYNJ2 are essential for anchoring Pink1 mRNA to mitochondria through the RNA-binding domain in SYNJ2, thus activating mitophagy.	Promote	(86)
Cerebrum	Morinda officinalis oligosaccharides reduce depression-like behavior in hypertensive rats by upregulating Mfn2 expression and initiating mitophagy through the PI3K/Akt/mTOR pathway, thereby effectively eliminating damaged mitochondria in astrocytes.	Promote	(87)

PINK1, PTEN-induced putative kinase protein 1; GSNOR, S-nitroglutathione reductase; Atg 5, autophagy protein 5; SYNJ2BP, synaptojanin 2 binding protein; SYNJ2), synaptojanin 2; Mfn2, mitofusin-2; SIRT3, PI3K, phosphoinositide 3-kinase; Akt, protein kinase B; mTOR, mechanistic target of rapamycin; sirtuin-3; ATF4, activating transcription factor 4.

### 3.1 Cardiac

Cardiac damage is a common type of organ damage caused by hypertension. A significant quantity of mitophagy is observable in a mouse model experiencing isoprenaline-induced cardiac remodeling. Interestingly, there was also a significant upregulation of mitophagy in the left ventricle of forkhead box protein P3 (FoxP3)-KO mice. Inhibition of FoxP3 expression and down-regulation of nuclear translocation leads to excessive Parkin-mediated mitophagy and more malignant cardiac remodelling. FoxP3 can downregulate activating transcription factor 4 (ATF4), decrease Parkin expression, and inhibit mitophagy, improving cardiac remodeling through two pathways (76). Dr. Tang isolated and cultured neonatal mouse cardiomyocytes from S-nitroglutathione reductase (GSNOR)-cKO mice. Mitophagy markers were detected. The results showed that the expression of PARKIN, PINK1, LC3II/I and p62 was significantly disorganized after Ang Ⅱ treatment, which was significantly ameliorated by overexpression of mtGSNOR, suggesting that mtGSNOR inhibits cardiomyocyte hypertrophy by regulating mitophagy. Furthermore, the researchers illustrated that GSNOR interacts with adenine nucleotide translocator isoform 1 (ANT1) to decrease SNO-ANT1 levels in mitochondria, encourage mitophagy, and improve mitochondrial dysfunction in hypertrophic cardiomyocytes (77). Dr Wei conducted a study on sirtuin-3 (SIRT3)-KO mice whereby they were treated with Ang II. The results of the study showed the presence of hypoxia and microvascular reduction in the cardiac tissue of the mice. In addition, mitochondrial dysfunction and cardiac fibrosis were also observed. They conclude that SIRT3 may promote angiogenesis and thus ameliorate myocardial fibrosis by attenuating mitochondrial dysfunction caused by defective mitophagy (78). Obesity reduces mitochondrial protein and deoxyribonucleic acid content and expands mitochondrial self-degradation in hypertensive hearts. The coexistence of obesity and hypertension also contributes to myocardial fibrosis and left ventricular diastolic dysfunction. Mitochondrial function improves cardiac damage in individuals suffering from hypertension and obesity (89). Valsartan can regulate mitophagy-related genes to inhibit excessive mitophagy, decrease blood pressure, and improve ventricular hypertrophy (79). In addition, spermidine was found to inhibit the progression of heart failure by regulating mitophagy to decrease blood pressure and prevent cardiac hypertrophy and diastolic dysfunction in Dahl salt-sensitive hypertensive rats (80).

### 3.2 Vascular

Vascular damage is prevalent among patients with hypertension, and it constitutes a significant contributing factor to the development of hypertension. Defective mitochondrial function promotes autophagy in endothelial cells, leading to reduced migratory and vasculogenic capacity, vascular endothelial dysfunction, and ultimately resulting in hypertension (90). Oxidative stress enhances mitochondrial damage, which in turn leads to vascular dysfunction, a process that is an important mechanism in the pathogenesis of hypertension. Astaxanthin promotes mitophagy and biosynthesis by upregulating the expression of Pink, Parkin, and mtDNA, resulting in restored mitochondrial function and improved vascular remodeling (81). It is likely that the decrease in angiogenic capacity of late EPCs, mediated by mitochondrial dysfunction due to defective CXCR4/JAK2/SIRT5 signal pathway, is responsible for capillary rarefaction in hypertension (91). Significant mitochondrial dysfunction was observed in rat aortic vascular smooth muscle cells treated with angiotensin II. This included increased oxygen consumption, elevated levels of ROS, reduced ATP production, decreased mtDNA levels, upregulated expression of Parkin and dynamin 1-like protein 1 (Drp1), and increased expression of peroxisome proliferator-activated receptor-γ coactivator-1α (PGC-1α) and transcription factor A (TFAM). Astragaloside IV has the potential to reverse the described phenomena. Based on the results, it can be inferred that Astragaloside IV is able to ameliorate the injury to vascular smooth muscle cells caused by Ang Ⅱ-induced mitochondrial dysfunction in rat aortic cells by promoting mitophagy and biosynthesis (92). Another study has shown that overexpression of SIRT3 enhances mitophagy for angiogenesis and ameliorates cardiac remodeling in hypertension (78). Increased division of endothelial mitochondria induced by Drp1 upregulation may be an important mechanism mediating vascular dysfunction in hypertension. Physical exercise or Drp1 inhibition effectively reduces mitochondrial division in endothelial cells, leading to the improvement of vascular endothelial function and the reduction of blood pressure (93). In mice injected with Ang II, Mito2HOBA enhanced Sirt3 activity and production of endothelial nitric oxide. It also reduced vascular superoxide levels, thereby improving endothelium-dependent relaxation, and decreased blood pressure. Furthermore, Mito2HOBA exhibited the ability to preserve mitochondrial respiration, protect ATP production, and reduce the opening of mitochondrial permeability pores in these Ang Ⅱ-injected mice (94).

### 3.3 Kidney

Hypertension elevates the risk of developing tubulointerstitial fibrosis, tubular atrophy, and glomerulosclerosis (95). The SIRT2/Septin4 deacetylase pathway mitigates hypertensive kidney injury by improving oxidative stress and preventing apoptosis in renal peduncle cells (96). Stimulator of interferon genes (STING) regulates renal inflammatory response and fibrosis induced by hypertension through acyl-CoA synthetase long chain family member 4 (ACSL4)-mediated fibroblasts. The inhibitors of ACSL4, rosiglitazone, and fibroblasts inhibitor, Fer-1, downregulated mtDNA/STING dependent renal inflammation induced by Ang Ⅱ (97). Adiporon, a lipocalin receptor agonist, improves renal fibrosis by promoting autophagy in a mouse model of hypertension induced by a high salt diet (82). Alfonso Eirin's team analyzed biological samples from 25 patients diagnosed with primary hypertension, 34 patients diagnosed with secondary hypertension caused by renal vascular disease, and 22 healthy volunteers. The findings indicate that blood pressure, urinary neutrophil gelatinase protein, and kidney injury molecule-1 levels were elevated in patients with both essential hypertension and secondary hypertension caused by renal vascular disease. The researchers concluded that heightened urinary mitochondrial DNA copy number may serve as an indicator for hypertensive patients and those with renal impairment and insufficiency. This indicates that there is mitochondrial impairment in hypertensive kidney disease in human (98).

### 3.4 Cerebrum

Hypertension represents a substantial risk factor for various cerebrovascular conditions, such as stroke and vascular dementia. Mitochondrial quality is crucial for the survival of neurons following ischemic insults (99). One study discovered that mitochondria located in the brain tissue of spontaneously hypertensive rats had a decreased ability to produce ATP (100). Another study indicated that losartan enhanced the metabolic of mitochondria in the brain tissue of spontaneously hypertensive rats (101). Mitophagy deficiency has been associated with various neurological disorders (102). Dr. Maurizio found that the brains of spontaneously hypertensive rats on a high-salt diet display impaired mitochondrial function. Mitophagy activation was found to reduce stroke risk (83). Chronic cerebral hypoperfusion is thought to severely affect cognitive function. Impaired autophagy or BNIP3-mediated mitophagy may serve as a mechanism for neuronal cell injury during chronic hypoxia (84). Interestingly, the process of mitophagy in ischemic neurons does not occur directly in the axon but instead takes place during the transit back to the neuronal cytosol. By activating mitophagy, damaged mitochondria are removed from neurons, ultimately providing protection (85). Interestingly, another study has indicated that synaptojanin 2 binding protein (SYNJ2BP) and synaptojanin 2 (SYNJ2) are required to tether Pink1 mRNA to mitochondria via the RNA-binding domain in SYNJ2 to activate mitophagy (86). More than half of individuals with hypertension suffer from depression (103). A recent study discovered that morinda officinalis oligosaccharides decrease depression-like behavior in hypertensive rats by increasing Mfn2 expression and initiating mitophagy through the PI3K/Akt/mTOR pathway, effectively removing damaged mitochondria in astrocytes (87). Moreover, research on hypertension induced by stress has indicated that inhibiting the HMGB1/RAGE axis increases stress-induced mitochondrial autophagic flux and reduces microglia-mediated neuroinflammation, which reduces sympathetic vasoconstriction in the ventral lateral aspect of the medulla cephaladis and decreases blood pressure (104).

## 4 Discussion

The projected prevalence of hypertension suggests that it will affect 1/3 of the global population until 2025 (105). Organ damage resulting from hypertension is a significant issue that requires attention. Hypertension is tightly linked to energy metabolism, and maintaining mitochondrial homeostasis is essential for the prevention and treatment of cardiovascular disease. A significant amount of evidence indicates that mitochondrial dysfunction is strongly related to HMOD. Mitophagy, an indispensable pathway for maintaining mitochondrial quality control, plays a significant role in hypertension and HMOD, such as improving cardiac remodelling, renal fibrosis, vascular remodelling, and nerve cell damage due to hypertension. Regulation of mitophagy has a positive impact on hypertension treatment. The PINK1/Parkin-mediated pathway for mitophagy is presently one of the most comprehensively researched pathways. PINK1 kinase activity and its localization to mitochondria are necessary for inducing Parkin translocation to depolarized mitochondria. In addition, the process by which p62 is recruited to the OMM is essential for mitophagy (106). It is apparent that PINK1/Parkin plays an extremely important role in mitophagy. Furthermore, PINK1 has the ability to regulate mitophagy-independent mitochondrial dynamics through phosphorylates Drp1 S616 (107). In the absence of PINK1/Parkin, a variety of receptor proteins exist to mediate mitophagy, such as BNIP3, NIX, FUNDC1, and FKBP8.

Typically, mitophagy is regarded as a safeguard or adaptive mechanism in pathological settings. Nevertheless, in certain instances, mitophagy can have negative effects. Maintaining cellular homeostasis relies on a delicate balance in mitophagy. Swift and effective removal of damaged mitochondria is imperative to prevent the buildup of dysfunctional organelles that may disrupt cellular function. Therefore, it is of paramount importance to comprehend the regulatory mechanisms and factors influencing mitophagy activity. In summary, two potential scenarios of mitophagy exist in hypertensive states: one displaying deficient mitophagy and the other exhibiting excessive mitophagy. Both scenarios prove detrimental to organismal homeostasis. Deficient mitophagy leads to excessive accumulation of mtROS and reduction of mtDNA, which in turn impairs mitochondrial and intracellular homeostasis. When excessive mitophagy occurs, the number of normal mitochondria in the cell decreases, resulting in a shortage in the cellular energy supply chain. Most of the current evidence suggests that promoting mitophagy reduces organ or tissue damage caused by hypertension. Nonetheless, other studies have found that inhibiting excessive mitophagy may have a protective effect. These two completely contrasting phenomena may be correlated with the disease type and stage. Therefore, it is imperative to solve this enigma as soon as possible. And that is also the direction of our research. The regulation of mitophagy is incredibly complicated, and its significance in disease is of utmost importance. The investigation of molecular mechanisms that underlie mitophagy in disease progression presents an auspicious approach for creating innovative therapeutic methods. This necessitates understanding the process involved, including its underlying biological principles, to combine with such mechanisms. Moreover, the clinical implementation of these findings demands meticulous assessment of their applicability in a clinical milieu.

Despite the large number of meaningful studies on mitophagy conducted by scientists, there are still many limitations. Firstly, mitophagy is a dynamic process, and most of the current studies only observe the changes in mitophagy at a certain point in time or over a period of time, which does not fully show the development of mitophagy in an organism. This is also related to the current detection technology. Secondly, mitophagy may have opposite effects in different diseases or at different stages of the same disease, which makes the study more difficult. Finally, most of the current studies are still at the stage of basic research, and translating basic research into clinical therapeutic strategies requires a more in-depth understanding of the mechanism of mitophagy in HMOD. Although many effective antihypertensive medications are widely available, the rate of control of hypertension remains unsatisfactory. Uncontrolled hypertension results in damage to multiple target organs. The development of artificial intelligence has greatly facilitated our work. Perhaps we can use AI to discover more gene targets that play a role in mitophagy, and then screen for mitochondria-targeted drugs to treat HMOD, or develop novel nanomaterials to deliver drugs to mitochondria. In addition, searching for reliable biomarkers of mitophagy in HMOD patients and developing non-invasive methods to assess mitochondrial autophagic activity could be used for early diagnosis and monitoring of treatment effects. All these are yet to be explored and investigated.
